# An improved high-performance liquid chromatography (HPLC) method for detection of variations in the hydroxyproline content of tissue homogenates from *Paracoccidioides brasiliensis*-infected mice

**DOI:** 10.1590/1678-9199-JVATITD-2023-0068

**Published:** 2024-07-05

**Authors:** Magnus Ake Gidlund, Raphael Fagnani Sanchez Molina, Eva Burger

**Affiliations:** 1 Department of Immunology, Institute of Biomedical Sciences (ICB), University of São Paulo (USP), São Paulo, SP, Brazil.; 2 Department of Microbiology and Immunology, Institute of Biomedical Sciences, Federal University of Alfenas, Alfenas, MG, Brazil.

**Keywords:** Paracoccidioidomycosis, Granulomatous lesions, Hydroxyproline, Collagen.

## Abstract

**Background:**

Paracoccidioidomycosis (PCM) is a severe granulomatous disease*.* The hallmark of this mycosis is fibrin degradation and granuloma formation as a result of a wound-healing process in the context of excessive inflammation. Therefore, as the content of collagen can be assessed by the methodology described in this manuscript, we propose that the content of hydroxyproline (HYP) be employed as a new and efficient measurement of granulomatous lesions developed. To estimate the level of HYP the major byproduct of the degradation process, we hypothesized that this simple and efficient technique could serve as a marker of disease severity.

**Methods:**

Five B10.A female mice were infected with *P*. *brasiliensis* and, after 15 days, the omentum was removed, subjected to histopathological analysis or processed (i.e. deproteinized and derivatized), and further analyzed on a reverse phase HPLC using a C-18 column. The omentum of five uninfected controls was also collected and similarly analyzed.

**Results:**

Infected mice showed numerous, disseminated paracoccidioidomycotic lesions, as well as marked collagen deposits, as observed in histopathologic analysis, and high levels of HYP. Normal uninfected mice showed no granulomas, little or no deposits of collagen fibers, and very low levels of HYP, as evaluated by HPLC. Our results show that the disease intensity as evaluated number and the morphology of the granulomatous lesions were correlated to the HYP levels using small tissue samples from the omentum, the main target organ of *P. brasiliensis*.

**Conclusions:**

Here we propose an alternative methodology to follow disease evolution and, to some extent, fungal load in experimental *P. brasiliensis* infection and suggest its usefulness to other diseases with pronounced fibrin degradation.

## Background

Paracoccidioidomycosis (PCM) is a severe systemic mycosis, caused by fungi of the genus *Paracoccidioides*. The disease is endemic in Latin America, and although its real prevalence is not known as compulsory notification is not required, it is considered a public health problem that affects mainly economically vulnerable populations involved in agricultural activities. It causes symptoms that threaten and compromise quality of life. Some of the problems of PCM are an intense inflammatory response and the difficulty of providing adequate therapy due to limited therapeutic choices. Immune response plays an important role in controlling the infectious process and the exacerbated inflammatory response that occurs in paracoccidioidomycosis.

Collagen is the main component of the connective tissue. It is a fibrous and tensile protein present both in the extracellular matrix and intracellularly, mainly in the skin and bones, as well as in tendons and cartilage. It is also present in the vessel walls conferring strength and elasticity. During pathological conditions such as skin fibrosis and bone remodeling, modifications of its concentration occur as a result of increased synthesis or degradation, and detection of such alterations can be useful for diagnostic and monitoring purposes. In this way, the quantification of the amino acid hydroxyproline (HYP) has been employed because 4-hydroxyproline represents about 14% of the total amino acid composition of collagen, being formed during the hydroxylation of proline after protein synthesis and released during collagen degradation. Different techniques were reported to detect this amino acid in biological fluids, mainly plasma and urine, such as spectrophotometric detection, electrophoresis, and chromatography. Considering the chemical characteristics of HYP, it is a secondary and non-essential amino acid, negatively charged at physiologic conditions. These properties make it possible to use chromatography to analyze the HYP content of biological samples. This technique is faster and more sensitive compared to the others and permits a variety of ways to prepare and analyze the samples, like gas chromatography, ion exchange, or reversed-phase liquid chromatography. In the great majority of cases, the sample preparation step is quite complicated, with requirements of long-time incubations at high temperatures and the use of chemical reagents that form unstable short-lived. 

Here we chose to work with a high-performance liquid chromatography **(**HPLC) method according to [1-4] modified to our conditions to test it for use as an indirect way to estimate the collagen content alterations in tissue homogenates during an experimental infection. The samples from omental tissues were obtained by an established protocol from control mice, inoculated with saline phosphate-buffered saline (PBS) or *P. brasiliensis*. Fungi of the genus *Paracoccidioides* cause paracoccidioidomycosis, a granulomatous disease [5]. Anatomopathological and histopathological studies on experimental Pb infection have shown the development of granulomatous lesions in the omentum, the target organ of *P*. *brasiliensis* in this experimental model, with distinct patterns of extracellular matrix components deposition [6, 7]. In resistant mice, two types of lesions were simultaneously observed: the first presented a well-defined encapsulated nodule, constituted mainly of type I collagen, and the other type showed residual characteristics, with sparse collagen deposits. In contrast, in susceptible mice, only one type of lesion was observed, showing less tendency to encapsulation and the formation of multiple small granulomatous foci, individualized by reticular type III collagen fibers. Altogether, the comparative histopathological analysis demonstrates the influence of the genetic pattern of the host on the lesions developed by resistant and susceptible mice to Pb infection.

Therefore, the role of the host’s genetic pattern on the granulomatous lesions developed after P. *brasiliensis* infection can be analyzed by histopathology. As the content of collagen can be assessed by the methodology described in this manuscript, we propose that the content of HYP be employed as a new and efficient measurement of granulomatous lesions developed and a prognosis for disease outcome.

## Methods 

### Animals

We used groups of five females, six to seven weeks old isogenic B10.A mice, susceptible to paracoccidioidomycosis [8], purchased from the Department of Immunology, Biomedical Sciences Institute, University of Sao Paulo animal facility. Five mice were infected and five mice were used as uninfected controls. The animals were kept in each cage, at a temperature of 22 °C receiving Nuvital chow and acidified water *ad libitum.*

### Fungus

The highly virulent Pb18 *P. brasiliensis* isolate [9] was employed for the infection. The fungi were kept in the yeast phase at 35-37 ºC by weekly sub-cultivations in Fava-Netto’s medium.

### Preparation of fungal suspension

The fungal growth was collected from the tubes at seven days of growth; the cells were washed three times with phosphate-buffered saline at pH 7.2, counted in a Neubauer hemocytometer chamber, and the concentration adjusted to 5x10^6^ yeast cells in a volume of 0.5 mL. Fungal preparations with 80% viability or higher as controlled with staining with Janus Green dye [10] were used for the infection. 

### Infection of mice

The intraperitoneal (IP) route was employed to inoculate groups of five mice and the samples were collected after 15 days. The experimental and control group consisted of five mice each.

### Collection of peritoneal omentum

Each animal was submitted to anesthesia with 20 mg/kg of the association tiletamine-zolazepam (Zoletil ®) by the intramuscular route [11] and then sacrificed. The procedures were performed according to standards recommended by the Ethics Committee for Animal Experiments at the University of Sao Paulo. The omentum was collected and weighed; part was used to assess the concentration of HYP; the remainder was used for assessment of granulomatous lesions by histological analysis.

### Preparation of the omentum for histological analysis

The omentum was fixed in Methacarn solution (60% methanol, 30% chloroform, and 10% acetic acid) for 3-4 hours in a shaker at 4 °C. Tissues were embedded in paraffin and five-micrometer sections were used in the histological analysis. Briefly, slide sections were deparaffinized and rehydrated, then stained with hematoxylin and eosin (HE), for visualization of the architecture of granuloma and with Picrosirius dye to identify the collagen fibers present in the organs through optical microscopy with the polarization of light [12]. The slides were mounted in Permout solution and observed in an optical microscope (Hund Wetzlar) with total increases of 40x, 100x, 250x, and 400x.

### Preparation of samples for hydroxyproline concentration determination

For this dosage, it was necessary to initially deproteinize the samples, which were added to 90% ethanol, under stirring for 10 minutes followed by centrifugation at 3000 xg for five minutes. The supernatant was collected and proceeded to evaporation of the sample in SpeedVac (Thermo Electron Corporation, model SPD131DDA) apparatus.

### Reagents for hydroxyproline concentration determination

To prepare the elution buffer, a 1M acetic acid solution (A) and a 1M sodium acetic acid aqueous solution (B) were prepared. The pH of the sodium acetate solution was adjusted to 6.5 through the addition of solution (A). Then this acetate buffer was diluted 50 times to the final concentration of 20 mM and the elution buffer was prepared by the addition of acetonitrile up to 36% and isopropyl alcohol up to 3%. Ortho-phthalaldehyde (OPA Sigma Aldrich) solution was prepared at 0.052 g/mL in methanol/ borate buffer 0.5 mol/L, pH 10.4/2-mercaptoethanol (8:30:1, by vol). Phenylisothiocyanate (PITC Sigma Aldrich) reagent was prepared with PITC/ethanol/MilliQ water/triethylamine (1:7:1:1, by vol). Acetonitrile, isopropyl alcohol, methanol, and ethanol were HPLC-grade from J.T. Baker. 

### Derivatization procedure

The lyophilized samples were reconstituted with 250 μL MilliQ water and derivatized according to the methodology described by Lange and Mályusz [1]. As HYP is a non-essential secondary amino acid, the primary amino acids were removed by the addition of 50μL of OPA solution to each 200 μL of the sample. After incubation for five minutes at room temperature, 200 μL of samples were passed through a SPE C18-E Stracta column (500 mg/3 mL - Phenomenex) previously activated with 3 mL of methanol and equilibrated with the elution buffer (sodium acetate 20 mM solution containing 36% of isopropanol and 3% of acetonitrile). Elution was carried out with 800 μL of elution buffer. The eluates were lyophilized and reconstituted with 800 μL of MilliQ water, to which 50 μL of PITC reagent was added to improve the HYP signal. After incubation for 20 minutes at room temperature, samples were lyophilized again and maintained at -70 °C until the HPLC analysis [2, 3].

### Chromatographic conditions

The samples were reconstituted with 800 μL of sodium acetate 20 mM, pH 6.5 solution, and filtered through a 0.45 μm membrane. These samples were manually injected in a C18-Novapack column (reversed phase; 3,9 mm × 15 cm; 4 μm; Waters) using a 20 μL looping. The HPLC system employed was a Shimadzu - Prominence model, with two LC-20AT pumps, and a diode array detector model SPD-M20A coupled to a CBM-20A communication unit. An isocratic elution was carried out using a sodium acetate 20 mM, pH 6.5 containing 36% isopropyl alcohol and 3% acetonitrile solution, at a flow rate of 0,4 mL/min. For the analysis, the wavelength was adjusted to 210 nm. An HYP standard (L-4-hydroxyproline) was used to determine the retention time and to make a standard curve with 5, 10, 25, 50, and 100 mM concentrations. The standard curve equation was obtained through linear regression data analysis and used to calculate the concentration of HYP in the samples.

### Chromatographic conditions

**Figure 1.**
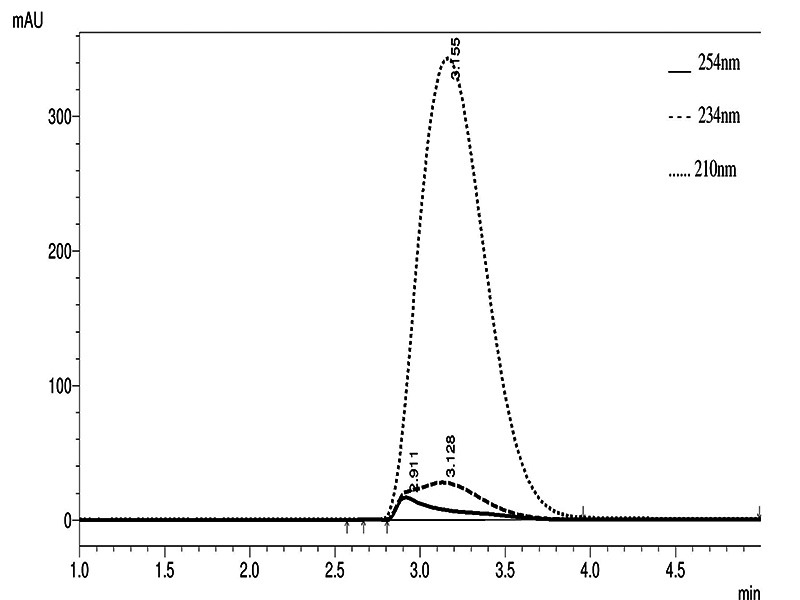
HPLC separation of L-4-hydroxyproline. In this experiment, a 20 μL aliquot of a 100 mM L-4-hydroxyproline standard solution was subjected to high-performance liquid chromatography (HPLC) using a C18-Novapack column. The elution conditions included a mobile phase consisting of a 20 mM sodium acetate buffer with 36% acetonitrile (ACN) and 3% isopropanol (pH 6.5), flowing at a rate of 0.4 mL/min. The absorbance of the sample was monitored at wavelengths of 210, 234, and 254 nm. Detailed information regarding the experimental conditions can be found in the Materials and Methods section.

The chromatographic procedure was based on the work done by Lange and Mályusz [1] with the following modifications. The pH was fixed at 6.5. To solve the reproducibility problems, the mobile phase concentration was reduced to 20 mM with the addition of 36% acetonitrile and 3% isopropanol (v/v). Because the addition of isopropanol increased the system pressure, the flow rate was reduced to 0.4 mL/min. In Figure 1 we applied a 100 mM L-4-hydroxyproline standard and we observed the peaks at 210, 234, and 254 nm. Under such a condition we obtained a major peak at 210 nm with minor contaminating peaks at 234 and 254 nm.

**Figure 2.**
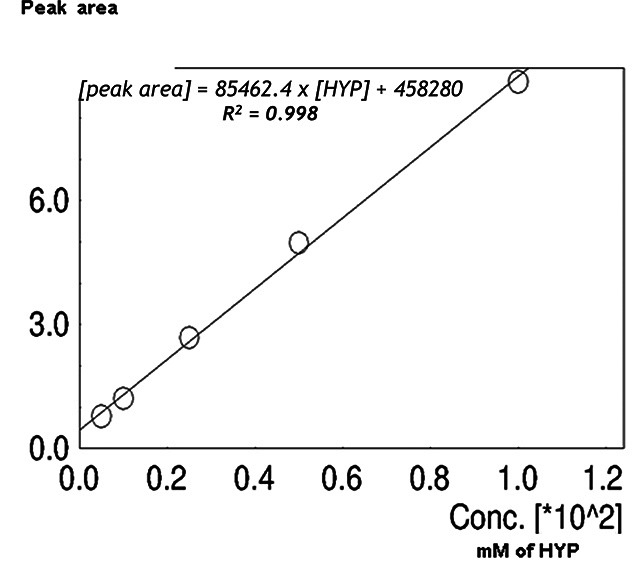
L-4-hydroxyproline standard-curve. To generate a standard curve, HPLC analysis was performed using a C18-Novapack column with L-4-hydroxyproline standard solutions at concentrations of 100, 50, 25, 10, and 5 mM. The analysis was carried out using the same elution conditions as the samples, which involved a 36% ACN, 3% isopropanol, 20 mM sodium acetate buffer (pH 6.5), and a flow rate of 0.4 mL/min. The absorbance of the samples was monitored at a wavelength of 210 nm. The retention time and peak area were calculated using the LC Solutions software provided by the manufacturer.

**Figure 3.**
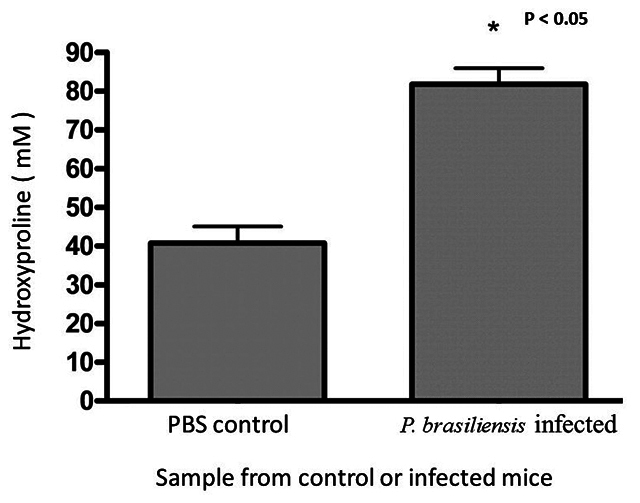
Quantification of HYP content of omentum homogenates. To determine the HYP content in omentum homogenates, samples from both **(A)** PBS-treated and **(B)** Pb-infected mice were OPA/PITC derivatized and injected into a C18-Novapack column. The mobile phase used was a 36% ACN, 3% isopropanol, and 20 mM sodium acetate buffer (pH 6.5), flowing at a rate of 0.4 mL/min. The absorbance of the samples was monitored at 210 nm. The retention time and peak area were calculated using the LC Solutions software provided by the equipment. The concentration of HYP was determined using the standard curve generated in Figure 2. Statistical analysis was performed using two-way ANOVA, and a p-value of less than 0.05 was considered statistically significant. Further details can be found in the Materials and Methods section.

To know the retention time of HYP, we make a standard curve by analyzing an external standard (L-4-hydroxyproline) at 100, 50, 25, 10, and 5 mM concentrations employing the established elution conditions. The absorbance values at 210 nm varied directly according to the standard concentration as can be seen in Figure 2. The linear regression analysis of these data showed good fitness of the curve (r^2^ = 0.997). The equation obtained was y = 85462.4*x + 45828, where ‘y’ is the peak area and ‘x’ is the HYP concentration in the sample. The data shown in Figure 3 indicates that the HYP content of the samples from normal PBS-treated mice was reduced compared to the HYP content of the samples from the *P. brasiliensis*-infected animals.

### Statistical analysis

The two-way ANOVA analysis was performed and results were considered statistically significant when p < 0.05 as indicated in the manuscript and Figure 3.

## Results 

To obtain reproducibility and avoid salt contamination of the column during the separation, we lowered the sodium acetate concentration, and the separation was performed with 20 mM Sodium Acetate containing 36% isopropyl alcohol and 4% acetonitrile 36 and 4% respectively. 

These conditions significantly improved both the problem with column and reproducibility and therefore maintained throughout the study. 

Alterations of the HYP content were detected through chromatographic analysis of the samples.

### HYP content and the deposit of collagen fibers in omentum were in parallel

**Figure 4.**
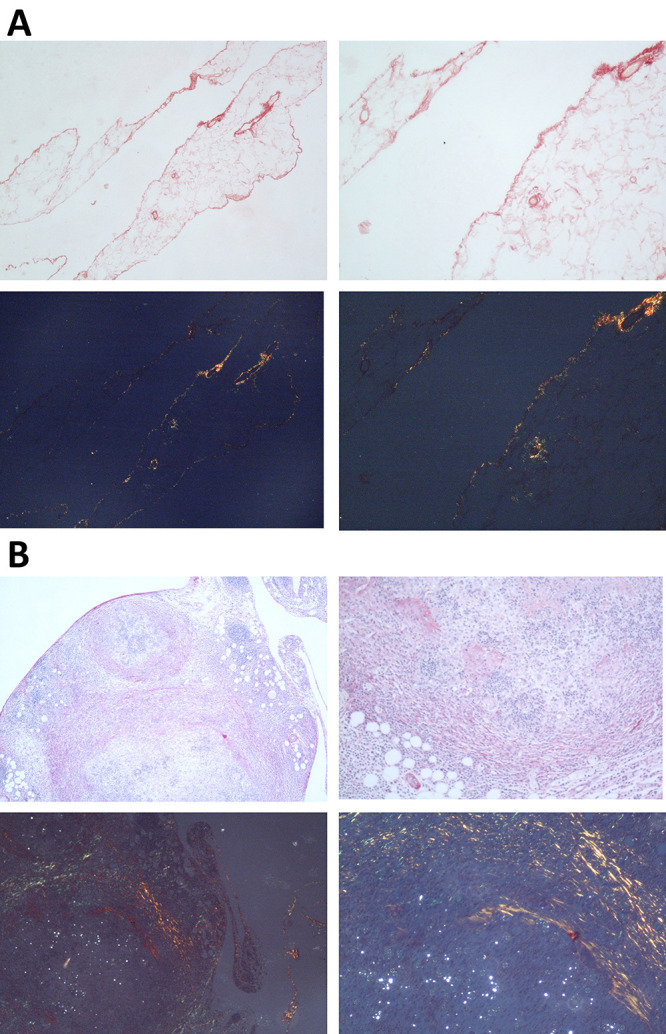
Microscopical detection of collagen fibers in the omentum tissue. Histological preparations of the omentum from (A) PBS-treated or (B) *P. brasiliensis*-infected mice were performed. The omentum was fixed in Methacarn solution for four hours at 4 °C. The fixed tissues were embedded in paraffin and five-micrometer sections were used in the histological analysis. Briefly, the slides sections were deparaffinized, rehydrated, and stained with hematoxylin and eosin (HE), for visualization of the architecture of granuloma (Figure 4A  and Figure 4B  upper panels) and with Picrosirius dye to identify the collagen fibers present in the organs through optical microscopy with polarization of light (Figure 4A  and Figure 4B  lower panels) The slides were mounted in Permout solution and observed using an optical microscope (Hund Wetzlar) at magnifications of 40x, 100x, 250x and 400x. under normal light to analyze the granuloma architecture (upper panels) and polarized light (lower panel) to make an optical estimate of the collagen deposits in the biopsies. For further details see Material and Methods.

To test the efficiency of the HPLC method to quantify the HYP content of our samples, the deposition of collagen fibers deposited on the omentum was evaluated through histology. Figure 4 shows histological preparations of the omentum from normal (A) and *P. brasiliensis*-infected (B) mice, stained with Syrius Red dye. Each preparation was analyzed microscopically under common light (1) and polarized light (2). Light microscopy examination evidenced the presence of numerous, disseminated granulomas, consistent with the pattern developed by susceptible mice after infection with *P. brasiliensis*. Comparing A and B it is possible to note that the preparations of normal mice tissue presented little or no deposits of collagen fibers in contrast with the *P. brasiliensis*-infected mice tissue, in all 3 microscopic analyses. At the same time, the preparations of these same tissues for chromatography showed the same collagen pattern of alterations in HYP content. In other words, preparations of omentum from normal mice had lower HYP content (39.27 ± 41.75 mM) than the preparations from *P. brasiliensis*-infected mice (82.81 ± 84.12 mM) (Figure 3). 

The results of microscopic analysis of stained tissue samples may eventually be influenced by subjective interpretations during the evaluation of the staining intensity of the structures, requiring an experienced technician as well as by technical artifacts, such as background staining. On the other hand, despite the HPLC technique requirements for high purity expensive organic solvents, it has a high sensitivity that permits the accurate quantification of less than 5 μM of a given molecule within small sample volumes (20 μL). 

## 4. Discussion

The major clinical event observed in PCM is an intense inflammatory response, with a conspicuous and persistent presence of polymorphonuclear neutrophils that are gradually substituted by macrophages differentiated to multinuclear giant cells and epithelioid cells, forming granulomas. These structures, which are dynamic and immunologically active, characterize paracoccidioidomycosis and present different morphological aspects depending on the evolution of the disease. When the mycoses are under control, both in the patients and experimental models, the granulomatous lesions are closed and the fungi are kept isolated by a thick layer of collagen. On the other hand, if the disease is out of control, the granulomatous lesions are open, disseminated, and do not restrain fungal dissemination. Thus, the pattern of collagen deposition is paramount to the outcome of this severe disease, and techniques that allow quantification of this phenomenon are extremely important to assay the clinical development that will follow, as well as the success of antifungal therapy.

The study of the *in situ* expression of cytokines in PCM granulomas allowed a better comprehension of the interaction of *P. brasiliensis* with the immune system at the granulomas is important. Burger et al. [13] associated the morphology of granulomas developed with the local synthesis of different cytokines. They showed that resistant mice that have compact granulomas develop a Th1-type response, with the presence of IFN-γ e TNF-α. This is in contrast to the susceptible mice which develop open granulomas and develop a Th2 response, with the presence of TGF-β.

Studies of matrix metalloproteinases (MMP) 2 and 9 in experimental PCM were also performed and demonstrated that MMP-2 has weak activity, but MMP-9 has high activity suggesting influence on granuloma and fungal dissemination. These authors also reported the immunolocalization of MMP-9 in multinucleated giant cells, macrophages, and lymphocytes present in the granulomas of *P. brasiliensis*-infected mice, suggesting that these cells are the main cellular sources of MMP-9. The presence and gelatinolytic activity of matrix metalloproteinases, particularly MMP-9 suggested their possible influence on the organizational pattern of the granulomatous lesions and also fungal dissemination in the same susceptible mouse strain as the one herein studied [14]. This information is in agreement with earlier data [6, 7] that detected multiple granulomatous lesions with less tendency toward confinement and marked the presence of type III collagen fibers with the increased production of HYP in infected susceptible mice here reported.

Therefore, the number and the morphology of the granulomatous lesions, as well as the presence of collagen fibers are directly involved in the outcome of paracoccidioidomycosis. In the IP model of PCM, extensively studied by our group, the histopathologic characteristics of severe paracoccidioidomycosis, which leads to bad outcomes, have been extensively characterized. Susceptible mice infected with the highly virulent Pb18 isolate of *P. brasiliensis* developed numerous disseminated granulomatous lesions which would result in an overall more marked presence of collagen fibers.

The present work focused on analyzing the collagen fiber deposits and the HYP content, comparing the observed patterns in normal versus infected samples. Histological analysis of Syrius Red-stained samples showed an increase in collagen fiber deposits in the omentum from infected versus the normal mice. Such an increase was a consequence of the induction of granulomatous lesions formation by the fungus, where an influx of inflammatory cells and the deposition of collagen fibers trying to restrain the infection is observed. Data from the HPLC analysis of the HYP content showed the same pattern of variation detected with the histological analysis of the collagen deposits, thus confirming the efficiency of the HPLC quantification of HYP as an estimative of collagen content. The HPLC methodology developed here we deem as a promising perspective as it represents an objective and highly sensitive technique that can be adapted to large sample numbers and small sample volumes.

## 5. Conclusions

The present work shows a much higher HYP content of omentum homogenates of *P. brasiliensis*-infected mice when compared to non-infected controls as soon as 15 days post-infection. This data is corroborated by histological analysis of Syrius Red-stained samples which shows an increase in collagen fiber deposits in the omentum from infected versus the normal mice simultaneously.

Considering the parallelism between the collagen content and the HYP concentration of the same samples (omentum), it is possible to assume that the HPLC technique is an efficient method for indirect estimation of collagen deposits, especially when dealing with small amounts of samples to search for molecules present at low concentrations.

The present simple methodology could be an alternative methodology to follow the evolution of some granulomatous diseases such as paracoccidioidomycosis and other diseases with pronounced fibrin degradation. As the presence of collagen fibers circumscribing the fungi, hydroxyproline quantification could, to some extent, indicate the fungal load in experimental *P. brasiliensis* infection. 

One of the most valuable pieces of information that the present work provides is that at a very early stage of infection (as soon as 15 days post-infection) there is a much higher HYP content of omentum homogenates of *P. brasiliensis*-infected mice as compared to non-infected controls. This data is corroborated by histological analysis of Syrius Red-stained samples which show an increase in collagen fiber deposits in the omentum from infected versus the normal mice

## Availability of data and materials

 All data generated or analyzed during this study are included in this article.
